# Lower Lobe, Higher Suspicion: An Atypical Presentation of Pulmonary Tuberculosis

**DOI:** 10.7759/cureus.93783

**Published:** 2025-10-03

**Authors:** Oscar Diaz, David Habib, Hector D Naranjo Valle, Lazaro Basart, Mariano Razzeto Rubio

**Affiliations:** 1 Dr. Kiran C. Patel College of Osteopathic Medicine, Nova Southeastern University, Fort Lauderdale, USA; 2 Department of Internal Medicine, Palmetto General Hospital, Hialeah, USA

**Keywords:** atypical radiographic findings, atypical tuberculosis presentation, lower lobe tuberculosis, occupational exposure to silica, pulmonary tuberculosis

## Abstract

Pulmonary tuberculosis (PTB) is a lower respiratory tract infection that most commonly presents as an upper lobe cavitation on imaging. However, atypical lesions, such as lower lung field tuberculosis (LLF-TB), can occur in some individuals, especially with underlying risk factors and comorbidities. We report a case of a 26-year-old male construction worker who presented with multiple episodes of hemoptysis, night sweats, high-grade fevers, and unintentional weight loss. Upon arrival, a complete medical history was taken, which was insignificant except for occupational exposure to silica particles. Initial chest radiographs (CXR) were unremarkable, prompting further evaluation with computed tomography (CT) of the chest, which revealed a necrotizing pneumonia along with ground-glass opacities in the left lower lung field. A rapid antigen detection test (RADT) for *Streptococcus pneumoniae* came back negative, and a clinical decision was made to empirically treat for bacterial pneumonia due to the severity of the CT results. Since the clinical suspicion for a tuberculosis (TB) infection was still high, a QuantiFERON-TB Gold test (QIAGEN N.V., Venlo, Netherlands) was ordered, which eventually came back positive. Following this, a bronchoscopy with bronchoalveolar lavage (BAL) was performed, and the presence of acid-fast bacilli (AFB) was confirmed. The patient was discharged home on standard RIPE (rifampin, isoniazid, pyrazinamide, ethambutol) therapy for six months and instructed to follow up with his primary care physician. This case highlights the atypical nature of this patient’s presentation regarding the location of their TB infection, as well as the absence of traditional risk factors associated with TB. In this case, we will discuss the diagnostic challenges associated with atypical TB cases and how occupational exposures may contribute to the onset, severity, or location of TB infection.

## Introduction

Pulmonary tuberculosis (PTB) has long been a significant public health issue, known for its contagious spread through the lungs and its various forms: latent, active, primary, and reactivated. A turning point came in 1882 when German microbiologist Robert Koch identified *Mycobacterium tuberculosis* as the cause of the disease [1,2]. His discovery marked a breakthrough in medical microbiology and was later recognized with the Nobel Prize in 1905. Around that same time, Franz Ziehl and Friedrich Neelsen developed a staining method using carbol fuchsin and nitric acid to detect the bacterium under a microscope. This method, now called the Ziehl-Neelsen (ZN) stain, became, and remains, a standard tool in diagnosing TB [3]. These early advances improved our understanding of the disease and helped shape the clinical practices used today to diagnose and manage TB worldwide. 

Chest radiography (CXR) is a standard initial imaging modality when diagnosing a patient with suspected pulmonary TB. In active pulmonary TB cases, common findings on radiography may show a tree-in-bud pattern, centrilobular nodules, consolidation, thick-walled cavities, or pleural effusions [4]. While these findings are frequently observed, this does not imply that they will always be present. Recent literature has reported that PTB findings were only seen in 49% of primary and reactivated cases on CXR, but other imaging modalities, such as computed tomography (CT) scans, demonstrated findings consistent with TB in 91% of cases [4].

Although pulmonary TB most often presents as cavitations in the upper lobes, certain recent studies have shown atypical findings of TB. Some atypical findings reported in recent studies include TB affecting the lower lung fields (LLF). The study identified risk factors for this atypical presentation, including immunosuppression, such as HIV [5]. In another study, researchers suggested that a low C-reactive protein (CRP) and poor physical activity were associated with cavitary and lower lobe disease presentation, respectively [6]. 

When examining atypical presentations, it is important to consider cases where patients, like the one discussed, do not have a significant past medical history that could influence the typical presentation of TB. In this particular case, the CXR done in the emergency department (ED) returned negative results, which aligns with the atypical presentation of TB discussed earlier. 

While many theories exist on the nature of atypical TB presentations, one theory of interest is the association between TB and occupational exposure, which may explain the lower lobe involvement in this patient's case. The tendency of TB to affect the lower lobe raises the question of whether pulmonary silicosis influences this presentation.

## Case presentation

A 26-year-old Cuban male, who immigrated to the United States two years ago, with no significant past medical history, presented to the ED with an episode of hemoptysis, non-productive cough, night sweats, unintentional weight loss of 15 pounds, and a temperature of 102°F. The patient denied a prior history of TB, incarceration, homelessness, or any form of travel in the past. The patient's social history was significant for working in construction for nine years; he admitted to being exposed to dust particles of silica and aluminum.

**Figure 1:**
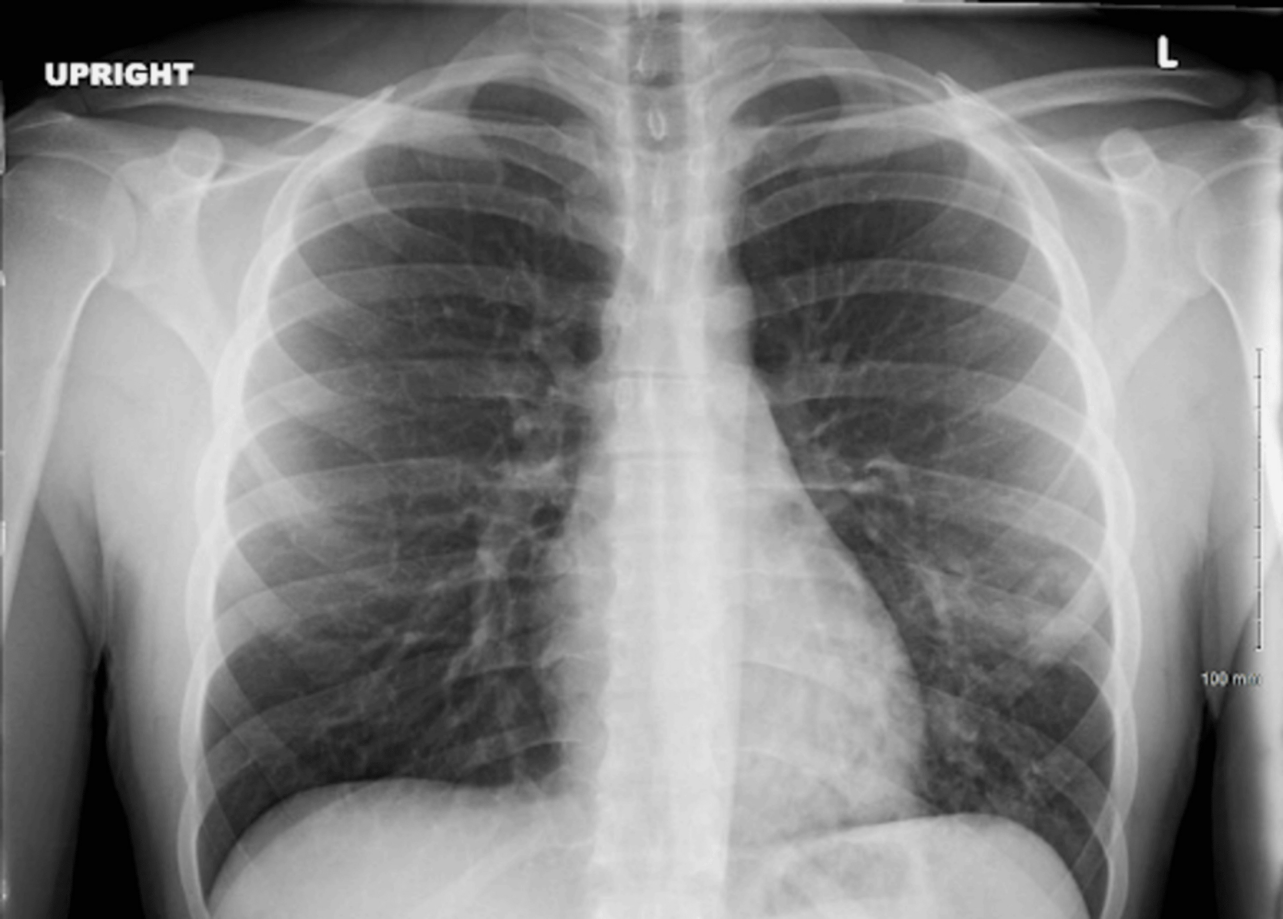
An upright anterior-posterior chest radiograph showing no remarkable abnormalities, such as infiltrates, consolidations, cavities, or lymphadenopathy associated with a pulmonary tuberculosis infection.

In the ED, an upright anterior-posterior CXR was taken, which came back negative, as shown in Figure 1. The patient was admitted for possible TB infection and systemic inflammatory response syndrome (SIRS)/sepsis criteria with pneumonia as a probable source of infection. Proper contact and isolation precautions were taken upon admission.

**Figure 2:**
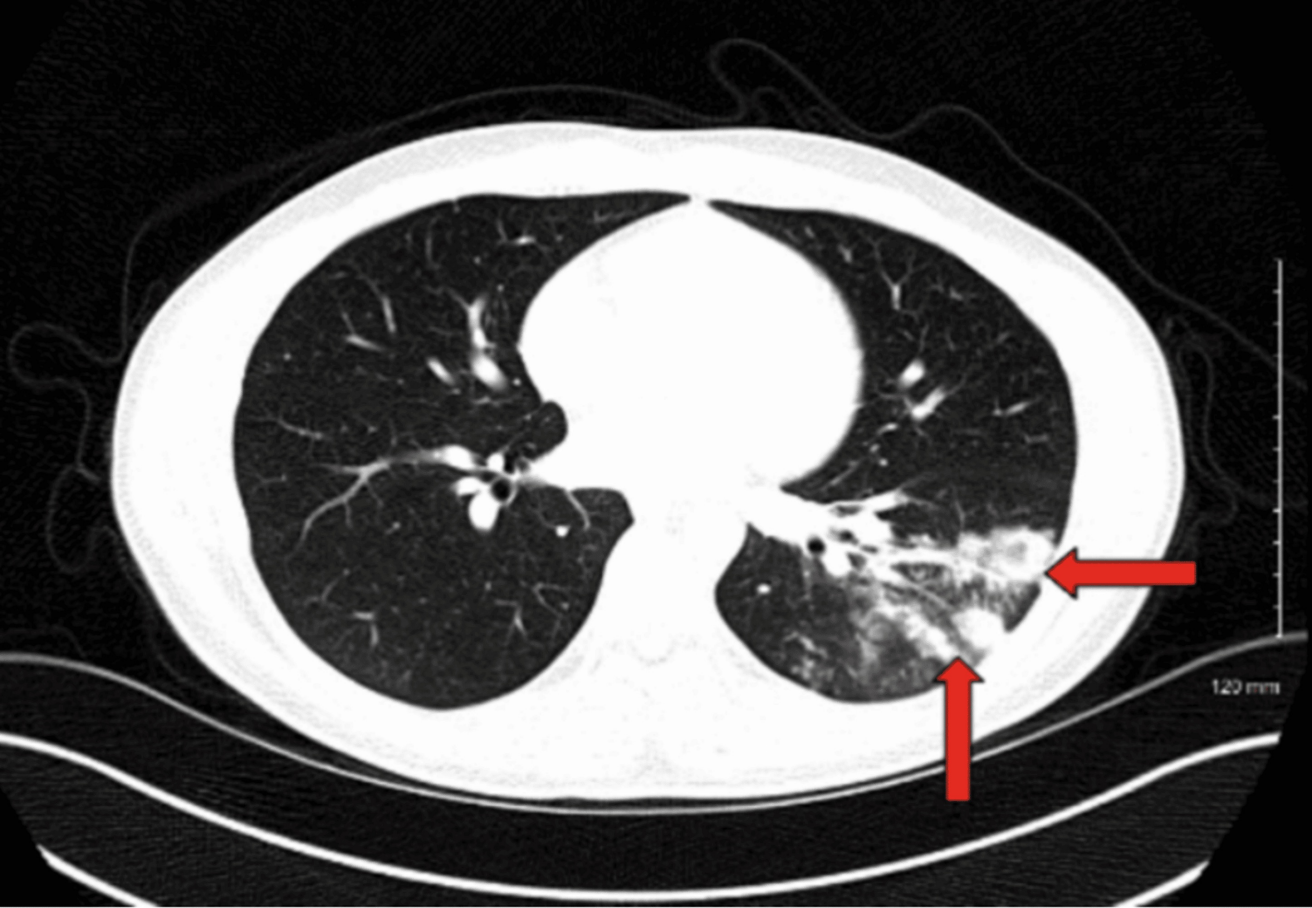
An axial computed tomography scan of the chest revealing cluster nodular and ground-glass density infiltrates with air bronchogram and necrotizing pneumonia affecting a significant portion of the left lower lobe as seen in the arrows.

**Figure 3:**
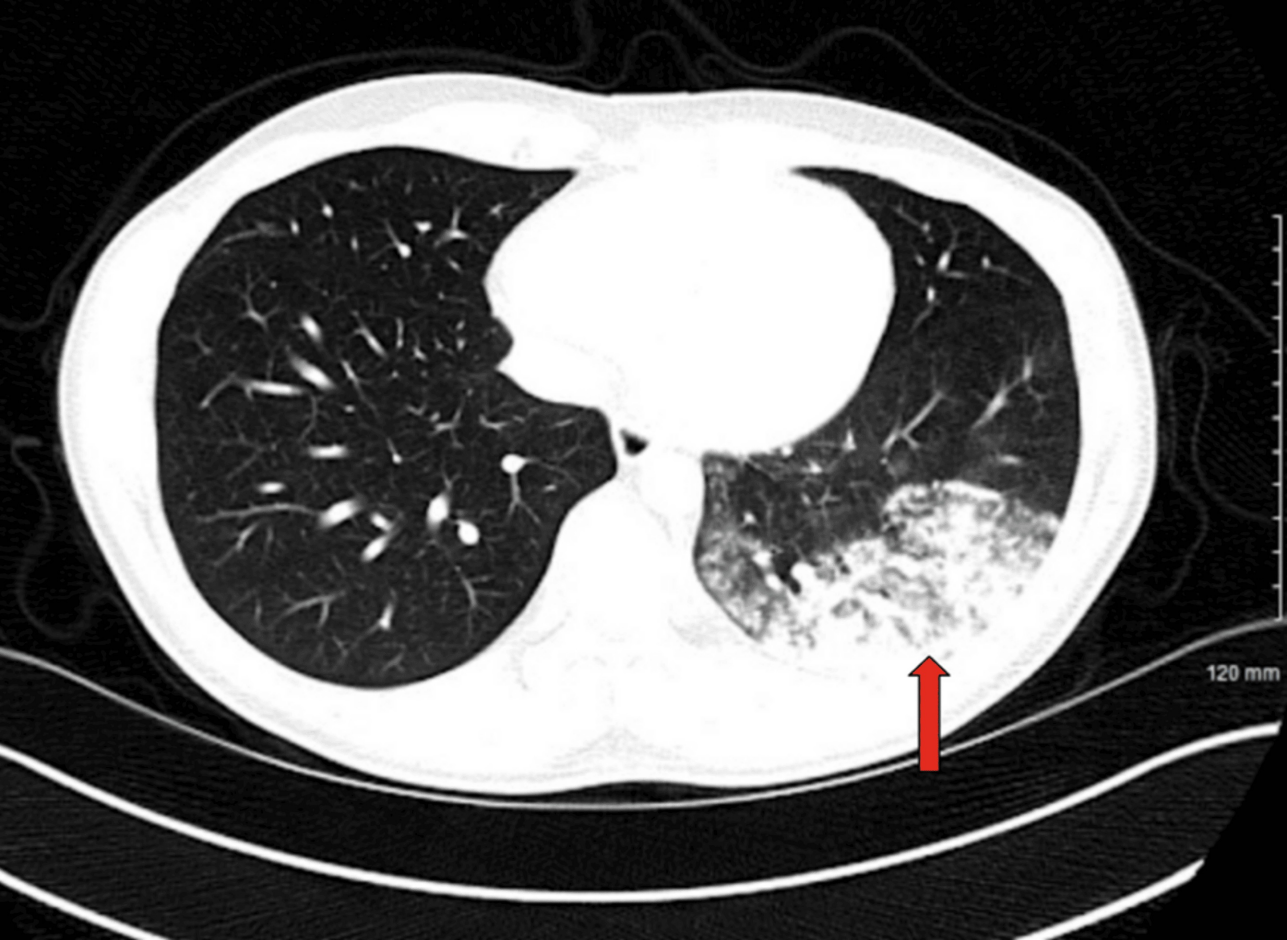
An axial computed tomography scan of the chest revealing cluster nodular and ground-glass density infiltrates with air bronchogram and necrotizing pneumonia affecting a significant portion of the left lower lobe as seen in the arrow.

An anti-neutrophil cytoplasmic antibody (ANCA) test was ordered due to the severity and sudden onset of symptoms, but it came back negative. A rapid antigen detection test (RADT) for *Streptococcus pneumoniae* returned negative. A CT scan of the chest was ordered and revealed a cluster of nodular and ground-glass density infiltrates with air bronchogram and necrotizing pneumonia affecting a significant portion of the left lower lobe, as shown in Figures 2, 3. 

Based on the severity of the chest CT results and the negative RADT, the medical team decided to empirically treat for a possible *Pseudomonas aeruginosa* infection with azithromycin and Zosyn (piperacillin-tazobactam). A QuantiFERON-TB Gold test (QIAGEN N.V., Venlo, Netherlands) was ordered and came back positive.

On day 4 of admission, the patient underwent a successful bronchoscopy procedure with bronchoalveolar lavage (BAL), which further confirmed the presence of a TB infection with acid-fast positive bacilli on culture with no signs of resistance to rifampicin. The patient was discharged on standard tuberculosis RIPE (rifampin, isoniazid, pyrazinamide, ethambutol) therapy for six months and instructed to follow up with his primary care physician.

## Discussion

The discovery of *M. tuberculosis* dates back as early as the 19th century, with Robert Koch as the pioneer of this discovery [1]. Even in the modern world, diagnosing a patient with PTB remains a challenge when initial radiographic findings are negative and when traditional risk factors are not present. In this case, the patient was a young, immunocompetent individual with an occupational history significant for silica exposure who presented with a LLF necrotizing lesion on CT imaging. 

An important concern in this patient's case is the possibility of considering a subclinical silica-related lung injury. Certain studies have reported an increased risk of TB infections in silica-exposed individuals even in the absence of silicosis on imaging [7-9].

Another interesting point worth consideration is the ability of CXRs to detect atypical presentations of active TB lesions. It is worth noting that there should still be a high level of suspicion of an active TB diagnosis even when initial imaging studies come back negative, which should warrant the utilization of advanced imaging modalities such as computed tomography [4,10,11]. In this scenario, the patient was empirically treated for a possible *P. aeruginosa* infection due to the severity of the CT results. The patient was only properly diagnosed with an active TB infection once the bronchial lavage results following bronchoscopy were available. 

LLF-TB has been shown to be associated in populations with comorbidities such as HIV, renal failure, or diabetes mellitus, with an estimated incidence of 1-18% of TB patients [12-14]. A study conducted in Bangladesh reported an increased incidence of LLF-TB amongst individuals with diabetes and those older than 40 years old [12], while another study found an increased incidence of LLF-TB amongst immunocompromised individuals [13]. The current patient's case is remarkable due to their young age and lack of reported comorbidities. This case raises the question of whether physicians might need to heighten their clinical suspicion of LLF-TB in an individual regardless of their medical history. 

Besides having an unremarkable past medical history, this patient admitted to having occupational exposure to silica, which is still clinically significant even in the absence of a confirmed diagnosis of silicosis. The mechanism by which silica exposure increases susceptibility to TB involves impairment of alveolar macrophage function, leading to dysregulated cell death (apoptosis and necrosis) and consequent weakening of the host’s immune defenses. A meta-analysis published by Ehrlich et al. revealed an increased risk of TB infection over a range of exposures, despite not having confirmed silicosis [8]. Additionally, a study conducted in Israel, which also investigated the relationship between silica exposure and TB incidence, reported a three times greater risk of TB amongst exposed individuals [7]. Although this patient's exact degree of silica exposure was not quantified, it would be proper to assume that their risk of TB infection was still elevated, even given an unremarkable medical history. These collective findings support the conclusion that a patient's occupational history should be considered when PTB is a high differential diagnosis. 

The diagnostic course in this patient highlights the limitations of CXR in this case. Further, it emphasizes the importance of utilizing advanced imaging and microbiological confirmation, such as the CT scan and acid-fast bacillus (AFB) stain. Despite the presence of constitutional symptoms, which are commonly associated with TB infection (i.e., fever, night sweats, and hemoptysis), the initial CXR was found to be negative. This finding aligns with multiple sources in which the authors highlight chest radiography's limited ability to detect findings of TB in early stages, atypical presentations (i.e., LLF-TB), or in immunocompetent hosts [2,5,10]. 

Compared to CXR, CT imaging has been shown to remarkably outperform when detecting active PTB and characterization of lesions [4]. In this case, the findings of the CT imaging of the chest were pivotal in reaching a definitive diagnosis. Compared to the CXR, the CT revealed a necrotizing pneumonia with nodular and ground-glass infiltrates in the left lower lobe. Due to the severity of symptoms and the findings seen with CT imaging, the suspicion for an active TB infection was elevated, which then prompted further and specific microbiological testing. This step in the diagnostic process was a crucial turning point, which should serve as an example for physicians dealing with a similar situation.

When dealing with this patient, the clinical team decided to order a bronchoscopy with BAL rather than rapid smear methods due to the location of the lesion and the absence of sputum production. This procedure is a reliable method of detecting TB, especially in atypical cases or when conventional sputum methods, such as the Ziehl-Neelsen stain, are unremarkable [3,14]. In the case of LLF-TB, bronchoscopy with BAL sampling is essential to attain a proper diagnosis, for patients are more likely to present with negative sputum smear results from standard testing [15]. Although bronchoscopy was more time-consuming and invasive, it served as a final confirmatory test, highlighting its importance when initial imaging and other microbiological tests are insignificant or unreliable.

## Conclusions

This case underlines the atypical presentation of TB in the LLF, as well as the importance for clinicians to maintain suspicion of TB infections in patients even when traditional risk factors are not present. Furthermore, it highlights the limitations of CXRs in the setting of atypical TB presentations and the advantages of advanced imaging and molecular techniques. With these points considered, this report suggests a need for physicians to re-evaluate strategies when dealing with unremarkable initial diagnostics and to take note of a patient’s occupational history and how that may tie into their diagnosis.
